# Genetic Complexity and Quantitative Trait Loci Mapping of Yeast Morphological Traits 

**DOI:** 10.1371/journal.pgen.0030031

**Published:** 2007-02-23

**Authors:** Satoru Nogami, Yoshikazu Ohya, Gaël Yvert

**Affiliations:** 1 Department of Integrated Biosciences, Graduate School of Frontier Sciences, University of Tokyo, Kashiwa, Chiba, Japan; 2 Laboratoire de Biotechnologie et Bioprocédés, Institut National des Sciences Appliquées, Centre National de la Recherche Scientifique UMR5504, Toulouse, France; 3 Université de Lyon, Lyon, France; Université Lyon 1, Lyon, France; Laboratoire de Biologie Moléculaire de la Cellule, Institut National de la Recherche Agronomique, Centre National de la Recherche Scientifique, Ecole Normale Supérieure de Lyon, Lyon, France; IFR128 BioSciences Lyon-Gerland, Lyon, France; Yale University, United States of America

## Abstract

Functional genomics relies on two essential parameters: the sensitivity of phenotypic measures and the power to detect genomic perturbations that cause phenotypic variations. In model organisms, two types of perturbations are widely used. Artificial mutations can be introduced in virtually any gene and allow the systematic analysis of gene function via mutants fitness. Alternatively, natural genetic variations can be associated to particular phenotypes via genetic mapping. However, the access to genome manipulation and breeding provided by model organisms is sometimes counterbalanced by phenotyping limitations. Here we investigated the natural genetic diversity of Saccharomyces cerevisiae cellular morphology using a very sensitive high-throughput imaging platform. We quantified 501 morphological parameters in over 50,000 yeast cells from a cross between two wild-type divergent backgrounds. Extensive morphological differences were found between these backgrounds. The genetic architecture of the traits was complex, with evidence of both epistasis and transgressive segregation. We mapped quantitative trait loci (QTL) for 67 traits and discovered 364 correlations between traits segregation and inheritance of gene expression levels. We validated one QTL by the replacement of a single base in the genome. This study illustrates the natural diversity and complexity of cellular traits among natural yeast strains and provides an ideal framework for a genetical genomics dissection of multiple traits. Our results did not overlap with results previously obtained from systematic deletion strains, showing that both approaches are necessary for the functional exploration of genomes.

## Author Summary

A familiar face or a dog breed is easily recognized because morphology of individuals differs according to their genetic backgrounds. For single-cell organisms, morphology reduces to the shape and size of cellular features. Microbiologists noticed that the shape of S. cerevisiae cells (baker's yeast) differs from one strain to another, but these differences were usually described qualitatively. We used a high-throughput imaging platform to study the morphology of yeast cells when they divide. Cells were stained with three fluorescent dyes so that their periphery, their DNA, and their actin could be recognized, and their images were analysed by a specialized software program. Numerous morphological differences were found between two distant strains of S. cerevisiae. By crossing these two strains, we performed quantitative genetics: several loci controlling morphological variations were found on the genome, and correlations were made between gene expression and morphology changes. Using bioinformatics, we showed that the results obtained do not overlap with previous results obtained from yeast cells in which specific genes are deleted. The study, therefore, illustrates how mutagenesis and the use of natural genetic variations provide complementary knowledge.

## Introduction

Yeast genetics has long been powered by the ease of conducting genome manipulation and mutagenic screens. These experiments are usually performed on a restricted panel of laboratory strain backgrounds that serve as standards. Variability between backgrounds is often viewed as a problem that must be minimized by using nearly isogenic strains whenever possible. As an alternative approach, several recent studies have used wild-type strains from divergent backgrounds to identify regulators of specific phenotypes such as high-temperature growth [1], sporulation efficiency [2], drug response [3], telomere homeostasis [4], or global transcriptional regulations [5]. This approach, which relies on genome scans for quantitative trait loci (QTL), emerged after high-throughput genotyping was facilitated by oligonucleotide microarrays [6]. It offers an effective alternative to conventional yeast genetics by employing the natural genetic diversity of wild yeast strains [7,8], thereby furthering the study of natural genetic resources.

In addition, exploring strain-to-strain variation in yeast or other model systems is essential to our understanding of the regulation of complex traits [9]. For example, one yeast study described the complexity of a QTL containing three genes each contributing to phenotypic variation [1]. Another study mapped transcriptional regulators, estimating the proportion of *cis*- and *trans*-regulatory variations and providing evidence for “master” regulators [5]. Similar conclusions were later obtained from mouse and human [10,11]. Since then, QTL controlling gene expression (eQTL) are sometimes included in mapping designs (an approach sometimes referred to as “genetical genomics”) and can provide judicious prioritization of candidate genes [12]. Furthermore, multiple phenotypes are now being acquired from patients or agronomical organisms, and strategies for QTL mapping of multiple traits are being given increasing attention.

Functional annotations of genomes are highly dependent on the sensitivity and scale of phenotypic tests [13]. Consequently humans, as well as pet dogs or cats, provide an excellent example of a system for understanding physiological variations, in addition to studying disease mechanisms, because they benefit from receiving extensive medical care. However, the capacity to manipulate genomes of model organisms is essential, and batteries of phenotypic tests have been developed for most of them, including mouse [14] and yeast. One of the most sensitive methods used to detect phenotypic variation in yeast has been the high-throughput characterization of cellular morphology [15]. This method is based on triple fluorescent staining of fixed cells using concanavalin-A, 4′,6-diamidino-2-phenylindole (DAPI), and phalloidin to label the cell wall, DNA, and actin, respectively. Microscopy images are then automatically acquired and analysed to quantify simultaneously 501 parameters from hundreds of cells.

We have applied this phenotypic characterization to a cross between two wild-type divergent yeast strains, which had previously been used in the study of the segregation of gene expression traits [16]. As described below, we showed that yeast cellular morphology is a complex trait with evidence of both transgressive segregation and epistasis. Using previously published datasets, we identified QTL controlling numerous morphological traits and found correlations between morphological and gene expression traits. We applied a bioinformatic comparison of these results to the results obtained when phenotyping systematic deletion strains and showed that the two approaches are complementary.

## Results

### Strain-To-Strain Natural Variation of S. cerevisiae Cellular Morphology

**Figure 1:**
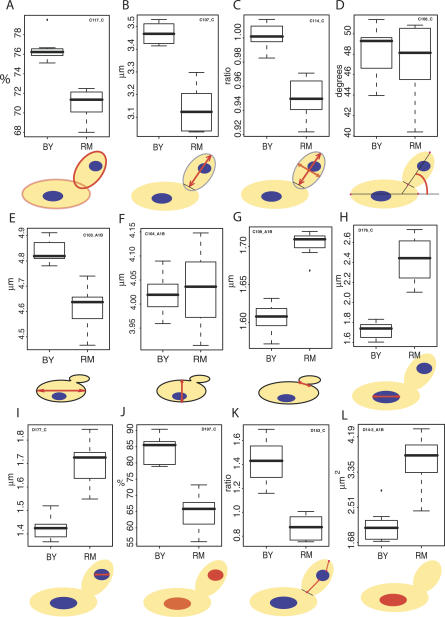
Representative Morphological Differences between BY and RM A quantitative comparison of BY and RM is shown for twelve phenotypes. The traits' definition is illustrated under each boxplot: (A) bud outline length relative to mother-cell outline length after nuclear division; (B) bud long-axis length after nuclear division; (C) bud axis ratio after nuclear division; (D) bud direction after nuclear division; (E) mother-cell long-axis length prior to nuclear division; (F) mother-cell short-axis length prior to nuclear division; (G) neck size prior to nuclear division; (H) mother-nucleus long-axis length after nuclear division; (I) bud-nucleus long-axis length; (J) ratio between bud nucleus size and mother nucleus size; (K) position of bud nucleus; and (L) DNA region size before nuclear division.

We looked for cellular morphological differences between laboratory strain BY4716 (isogenic to S228c) and strain YEF1946, isogenic to RM11-1a, which is a haploid derivative of a wine strain (kindly provided by E. Foss). The original RM11-1a could not be used because of its clumpy nature [17], which was suppressed in YEF1946 by a single base substitution in the *AMN1* gene. For simplicity, strains BY4716 and YEF1946 will, nonetheless, be referred to as BY and RM hereafter. Samples from nine independent cultures of each strain were characterized by triple-staining fluorescent microscopy and automated cell imaging as described previously [15]. At least 200 cells were analysed per culture to quantify 501 morphological parameters that were each considered as a quantitative trait (Table S1). For each trait, we tested the difference between the nine BY and the nine RM values using the Wilcoxon Mann-Whitney test. At *p* < 0.001, 143 traits showed significant difference. A permutation test determined that only one trait was expected to differ by chance at this *p*-value. The morphological differences between the two strains were found at different stages of the cell cycle and reflected various cellular aspects (Table 1). After nuclear division, buds from BY cells were bigger than those from RM cells (Figure 1A and 1B); they were also more elongated in BY (Figure 1C). No difference was seen in the direction of bud growth (Figure 1D). Mother cells from BY were more elongated (Figure 1E and 1F), bud necks were bigger in RM cells at early division (Figure 1G), and cell-wall thickness was more homogeneous in BY cells (Table 1). Surprisingly, a large majority of the differences corresponded to DNA staining patterns (106 of the 143 traits) (Figure 1H–1L; Table 1). However, DNA staining is covered by 272 of the 501 parameters estimated by CalMorph, which is a significant enrichment as compared to actin- or cell-wall–related parameters. In addition, using the dataset from Ohya et al. [15], we found that DNA-related traits had lower measurement errors than other traits (see Materials and Methods). Thus, our procedure seemed to have a better ability to detect differences in intracellular DNA distribution than differences in actin distribution or cell shape, possibly explaining the majority of DNA-related parameters in Table 1. In addition to phenotypic differences at specific stages of the cell cycle, we observed variation of the proportion of cells at particular stages. For example, the fraction of RM cells with small buds already containing DNA was high, whereas the progression of bud growth was similar in both strains. Similarly, the fraction of budded cells containing only one DNA region was small in RM. These observations could result from two phenomena: either premature nuclear division may occur in RM cells even when bud growth is inadequate, or the fraction of cells having passed nuclear division is overestimated in RM. In the former case, parameters reflecting actin organization should differ as well, because apical actin distribution begins in G1 and continues until beginning of nuclear division [18]. This was not the case, since we did not observe any change in the fractions of cells showing apical actin distribution over all stages (parameters A106 and A107). Alternatively, mitochondria were more abundant in RM cells when observed after MitoTrackerGreen labelling (unpublished data), and these organelles are known to move into the bud prior to the accomplishment of nuclear division [19]. It is, therefore, possible that mitochondria abundance or differences in their intracellular distribution bias the inventory of cells containing two nuclei.

**Table 1:**
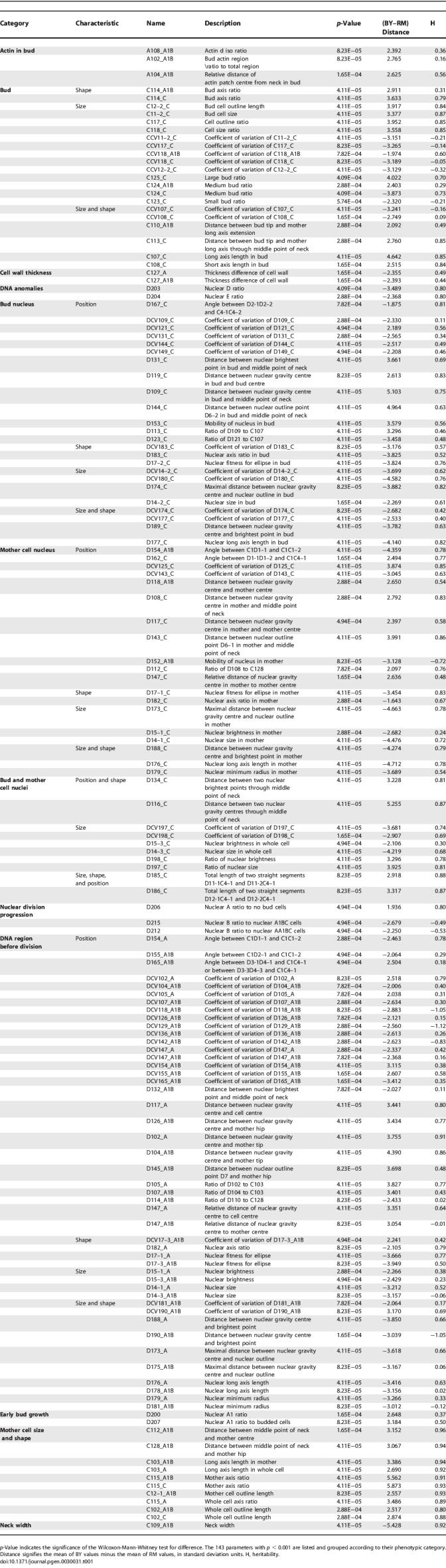
Morphological Differences between BY and RM Strains

### Complex Genetic Segregation of Yeast Morphological Traits

**Figure 2:**
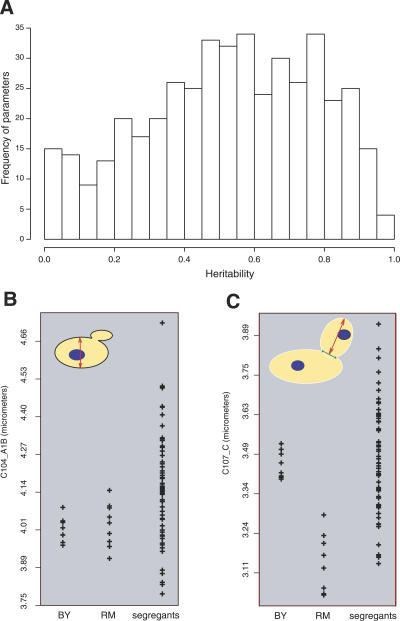
Genetic Segregation of Morphological Traits (A) Heritability of 501 morphological traits; 62 traits that had negative heritability (no detectable genetic variance) are not represented. (B) Example of transgressive segregation. Each cross represents one measurement of parameter C104_A1B (length of the short axis of the mother cell). The first and second series are replicate cultures of BY and RM, respectively. Each cross of the last series represents the average value of one segregant measured in triplicates. Many segregant values fall outside the parental range. The inset drawing illustrates the trait definition. (C) Example of both epistasis and transgressive segregation. Trait C107_C (length of the long axis of the bud) is represented as in (B). Segregants values are not centred at mid-parental value (epistasis), and many segregants show higher values than BY (transgression).

In a previous study, the segregation of gene expression levels was studied across 112 F1 segregants from BY × RM [16]. We used these F1 strains to study the genetic segregation of morphological differences between BY and RM. Of the 112 segregants provided, five were flocculent and 45 were clumpy. These strains were not suitable for image analysis of isolated cells and were therefore discarded. The remaining 62 strains were cultured in triplicates and over 200 cells per culture were processed to quantify 501 morphological parameters. Each parameter was then treated as a quantitative phenotype. Median heritability among all 501 parameters was 49%, with 71, 121, 146, and 101 showing low (0%–25%), moderate (25%–50%), significant (50%–75%), or high (>75%) heritability. This suggested that experimental errors were low enough to study the genetic control of the majority of the traits (Figure 2A). We noted that 62 phenotypes had negative heritability values (no detectable genetic variance), which either means that their measurement errors are too high to detect genetic control, or that the genes controlling them do not harbour functional variations between the BY and RM backgrounds. Intriguingly, 16 of those traits belonged to the list of 143 traits differing between BY and RM (Table 1), suggesting epigenetic control. Consistently, many of these traits might be affected by mitochondrial DNA distribution (for example, the “mobility-of-nucleus-in-mother-cell”). Differences in mitochondria abundance or spatial repartitions between BY and RM can explain “non-heritable” differences, since mitochondria are not inherited via Mendelian segregation but undergo a complex fusion process through meiosis [20].

To estimate the complexity of the genetic control, we looked for cases of transgressive segregation or epistasis. We applied tests previously adapted for multiple traits [16] and found that about one-fourth and two-fifths of all phenotypes showed transgressive segregation and epistasis, respectively. We detected 34 phenotypes significantly transgressive at a False Discovery Rate (FDR) of 0.05, and 98 phenotypes significantly epistatic at FDR = 0.05 (see Materials and Methods; Table S2 and Table S3). As examples, the segregation patterns of two such traits, the short axis length of the mother cell and the long axis length of the bud, are shown in Figure 2B and Figure 2C, respectively.

### QTL Mapping of Morphological Traits

**Figure 3:**
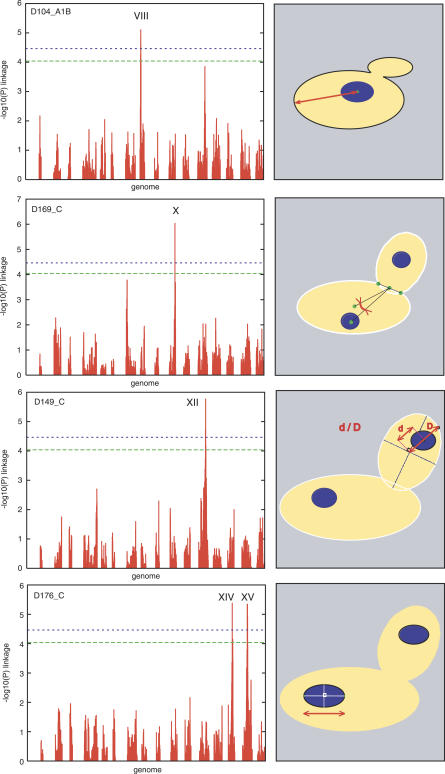
Examples of QTL Mapping of Morphological Traits Each plot on the left represents a genome scan mapping a morphological trait, where the *x*-axis is the physical position of the genetic markers, and the *y*-axis represents nominal *p*-value of the linkage test (plotted as −log10(*p*)). Each vertical bar represents the linkage result for one of the 3,042 genetic markers. Artificial gaps in the *x*-axis were added to distinguish consecutive chromosomes from one another. The two genome-wide significance thresholds mentioned in text are indicated by green and blue dashed lines. QTL chromosome number is indicated above significant peaks. Traits are indicated at the top left corner of the plot and their definition is represented in red on the right-hand side drawings. D104_A1B was the distance between the nuclear gravity centre and the tip of the mother cell; D169_C was the angle between the axis defined by the midpoint of bud neck and the centre of the mother-cell nucleus and the axis defined by the midpoint of the bud neck and the centre of the mother cell; D149_C was the ratio d/D corresponding to the distance between the centre of the bud and the centre of its nucleus, relative to the bud size; D176_C was the length of the long axis of the mother cell nucleus.

Using a genetic map of 3,042 markers previously generated by Brem et al. [16], we sought to map QTL of morphological traits. Of the 501 total morphological traits, 254 were discarded because they showed low or moderate (<50%) heritability. Of the remaining 247 traits, 95 and 67 could be mapped (i.e., at least one locus controlling their variation could be mapped) at *p* < 9.04 × 10^−5^ and *p* < 3.43 × 10^−5^, respectively. A permutation test determined that these *p*-values corresponded to FDR = 0.10 and FDR = 0.05, respectively. A total of seven distinct loci controlling specific morphological features were identified at FDR = 0.05 (Table 2; Table S4). When several phenotypes were mapped to the same locus, they were usually different estimators of the same cellular features (for example, “long-axis-length-in-mother” and “distance-between-middle-point-of-neck-and-mother-center” are two measurements of the mother cell elongation). The phenotypes were mapped for various aspects of cellular morphology: the localization and the shape of the DNA regions within the mother cell or the bud, the heterogeneity of the DNA staining, and the size or shape of the mother cell (Figure 3; Table S4). Surprisingly, 12 traits informative of the size, shape, and position of the DNA staining were mapped to two unlinked loci located on Chromosomes XIV and XV, respectively.

**Table 2:**
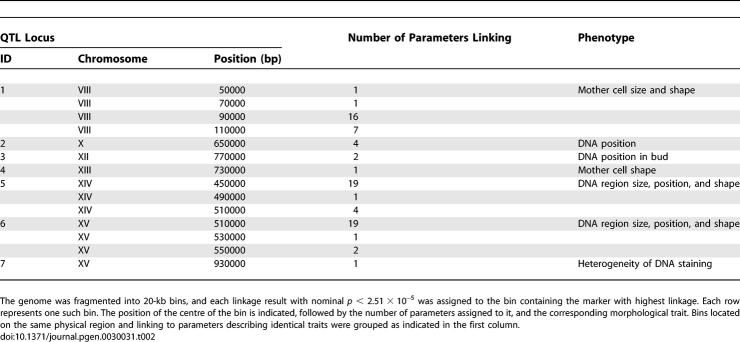
Morphological QTL (at FDR = 0.05)

### Genetic Correlation to Transcriptional Regulations

**Figure 4:**
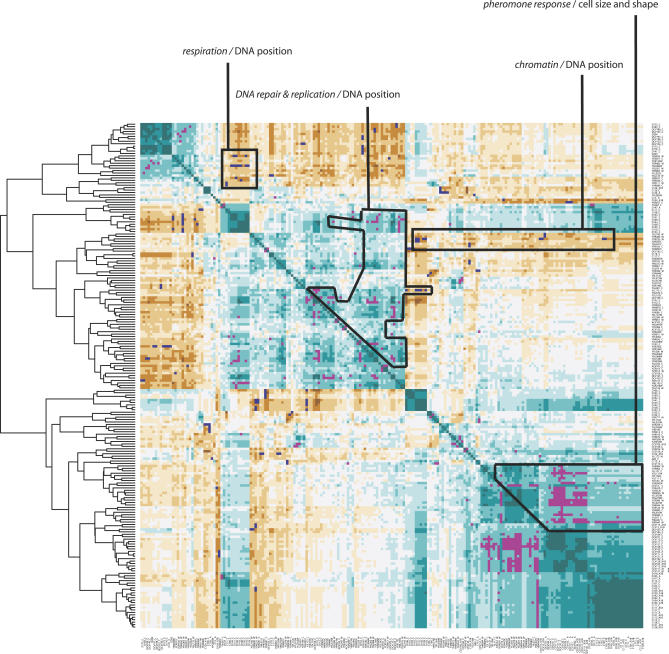
Genetic Correlation between Morphological Traits and Gene Expression The matrix shows the correlations among the expression levels of 103 genes and the values of 104 morphological traits. Brown and blue-green indicate negative and positive correlations, respectively. The set of significant correlations (FDR = 0.1) between morphological traits and gene expression levels described in text is indicated by blue and magenta dots, reflecting negative and positive correlations, respectively. For clarity, hierarchical clustering was performed and its result is indicated by the dendogramm. The four boxed regions are areas where magenta and blue dots covered a set of genes of similar function and a set of traits describing similar morphology.

In the study from Brem et al. [16], all 62 F1 strains were grown in the same conditions as here, and their expression profiles were determined on DNA microarrays. Using this dataset, we searched for genetic correlations between morphological trait values and gene expression levels by computing the absolute value of the Pearson correlation coefficient (|*R*|). Among 247 traits showing heritability greater than 50%, 104 and 29 could be correlated to the expression level of at least one gene at |*R*| > 0.565 and |*R*| > 0.62, respectively. A permutation test determined that these correlation values corresponded to FDR = 0.1 and FDR = 0.05, respectively. At FDR = 0.1, a total of 364 correlations involved the expression levels of 103 genes (Figure 4; Table S5). We found several cases where annotations of the genes involved were consistent with the correlated phenotypes. For example, expression levels of *FLO11* (YIR019C) and *ECM34* (YHL043W, involved in cell wall regulations) were correlated to brightness differences of the cell wall. To look more systematically for such consistencies, we clustered hierarchically the 104 traits and 103 genes involved, and examined territories of the correlation map containing several gene/traits correlations (Figure 4). We found four such territories where gene ontology (GO) annotations were indicative of a cellular process or component correlated to traits. Expression levels of *PET117* (YER058W), *SAL1* (YNL083W), *SCO1* (YBR037C), *YNR036C, DBF20* (YPR111w), and *YHR080C* were correlated to nine traits measuring the position of DNA in the mother cell and in the bud after nuclear division. This suggested a link between DNA positioning and the GO terms “protein metabolism” (4/6 genes, *p* = 0.02) and “mitochondrion” (5/6 genes, *p* = 0.00032). Since DNA positioning estimation can be affected by mitochondrial DNA staining, it is very likely that these variations in mitochondrial activities between BY and RM cells are associated with different regional distributions of mitochondrial DNA. Expression of 22 genes, including *TOP2* (YNL088W) and *MSH2* (YOL090W), were correlated to 18 traits also describing DNA positioning in mother cells and buds after nuclear division. These correlations linked these traits to many GO terms reflecting DNA metabolism, including “DNA-dependent DNA replication” (4/22 genes, *p* = 0.00014) and “DNA repair” (3/22 genes, *p* = 0.012). This association suggests that differences in nuclear DNA metabolism drive differences in DNA positioning. Expression levels of *SEH1* (YGL100W), *YHR200W, HTA1* (YDR225W), *GRD19* (YOR357C), and *HHT2* (YNL031C) were correlated to ten traits describing the position of DNA during cellular division, the size of the bud neck, and the position of DNA in the mother cell after nuclear division. These traits were therefore associated to GO terms “chromatin assembly or disassembly” (2/5 genes, *p* = 0.002). This suggests that differences in DNA intracellular distribution probably result from differences in chromatin dynamics throughout nuclear division. Finally, expression levels of 23 genes, many of which being involved in pheromone response, were correlated to 39 traits describing the mother cell shape (GO terms “conjugation” (17/23 genes, *p* < 10^−25^) and “response to pheromone” (15/23 genes, *p* < 10^−24^). This association strongly suggested that cell shape differences resulted from differences in the activation of the pheromone response pathway between the two genetic backgrounds. A direct explanation of this is given in the next section.

### A Non-Synonymous Single Nucleotide Polymorphism in GPA1 Affects Cell Elongation

**Figure 5:**
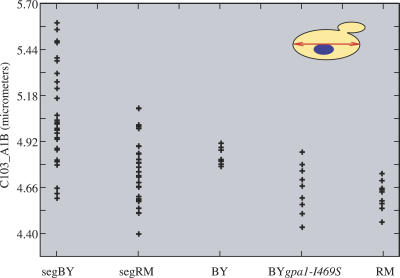
Validation of a Quantitative Trait Nucleotide in GPA1 Measurements of parameter C103_A1B (length of the long axis of the mother cell) are plotted along the *y*-axis. Each dot of the segBY and segRM series represents the average value of one segregant measured in triplicates. Segregants of segBY and segRM are the segregants having inherited *GPA1* from BY and RM, respectively. Each dot of the last three series represents one culture of the corresponding strain. The values of BY*gpa1-I469S* differed significantly from those of BY (Wilcoxon Mann-Whitney *p* = 0.002) and were comparable to the RM values.

We then sought to identify polymorphisms responsible for morphological differences. We focused on 16 cell-elongation traits linked to a locus on Chromosome VIII that contained the *GPA1* (YHR005C) gene (Table 2). A previous study showed that a single polymorphism in *GPA1*, S469I, was responsible for constitutive residual activation of pheromone response genes in BY [17]. Response to pheromone includes elongation of cells that prepare for mating (“shmoo” phenotype). This polymorphism was, therefore, an excellent candidate to explain the morphological QTL. To test the *GPA1-S469I* polymorphism for cell-elongation differences, we measured morphological phenotypes of BY-*gpa1I469S*, an engineered strain isogenic to BY except that it carried the RM allele of *GPA1* [21]. Of the 16 traits linking to *GPA1*, nine differed significantly between the nine replicates of BY and RM (*p* < 0.05), and seven of these differed accordingly between BY and BY-*gpa1I469S* (*p* < 0.05) (Table 3). Values for one trait, the long-axis length in the mother cell, are shown in Figure 5. The results demonstrated that the *GPA1-I469S* polymorphism was responsible for cell-elongation differences. The fact that several traits linked to the *GPA1* locus but were not significantly different between BY and RM (or between BY and BY-*gpa1I469S*) can be due to a reduced statistical power when comparing strains (2 × 9 values), as compared to linkage test (62 values), or to additional undetected QTL with opposing effects.

**Table 3:**
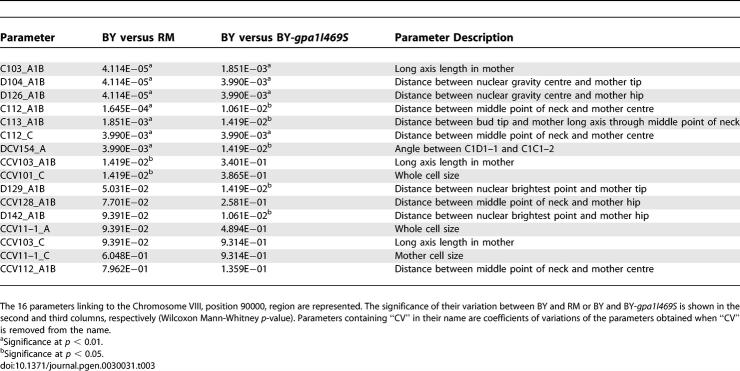
Validation of a Quantitative Trait Nucleotide in GPA1

### QTL and Deletion-Based Strategies Give Complementary Results

In a separate study, the cellular morphological alterations due to single gene disruptions were investigated in the context of the BY genetic background [15]. For all 4,718 nonessential genes of the genome, a haploid strain deleted for that gene was analysed using the same staining protocol and imaging platform as we used here, giving quantification of the same 501 morphological traits. We sought to compare the results from this systematic-deletion strategy to the results obtained in our quantitative genetics approach, addressing the following question: When a morphological trait T is mapped to a QTL, is there a gene in the neighbourhood of this QTL of which the deletion affects T? If the answer is yes, a polymorphism in this gene could explain the QTL mapping result. We found a single case where this overlap between the two datasets was observed, and we estimated that finding one such case by chance only was highly expected (see Materials and Methods). Therefore, the majority of QTL results cannot be “explained” merely by genetic variation occurring in genes previously identified from the deletion set.

## Discussion

We found extensive cellular morphological variations between two wild-type S. cerevisiae backgrounds. The appearance of yeast is therefore polymorphic, as is the appearance of two unrelated human beings or animals. Out of curiosity, we asked colleagues to distinguish BY4716 from YEF1946 liquid cultures under the microscope (light microscopy on living cells, 40× magnification). From their responses, it was obvious that no difference would have been characterized without the fluorescence staining and automated quantification that we used. We found significant QTL for only 27 of the 143 traits differing between the two backgrounds. Differences in actin distribution in buds, bud shape and size, early bud growth, thickness of the cell wall, or neck width, as well as most traits related to DNA distribution prior to nuclear division could not be correlated to genomic loci. This can result from the small heritability of some of the phenotypes, as mentioned above for parameters that can be affected by the abundance and distribution of mitochondria. It can also result from the high complexity of the genetic control, whereby many loci contribute to the phenotype, but their small individual effect prevents their detection. Conversely, 40 of the 67 traits for which QTL were found were not in the list of the significant differences between parental strains. This was the case for phenotype CCV115_C describing the variation of mother-cell axis ratio across the sample (mapped to Chromosome X), and phenotype DCV194_C describing signal heretogeneities in mother cell DNA (mapped to Chromosome XV 930000). There are several possible explanations for this: some of these linkage results might be false positives (which we would expect to be possible for one or two but certainly not 40 phenotypes), alleles acting in the opposite direction might shade their effects in the parental strains (which would be consistent with the extent of transgressive segregation), or simply because statistical power was sometimes higher during mapping (differences among 62 values in segregants instead of 18 in the parents).

Notably, only 12 out of the 67 traits for which significant linkage was found were mapped to more than one place of the genome. Considering the large extent of transgressive segregation and epistasis, it is very likely that other loci contribute to morphological variations, and detecting only one or two per trait is probably a statistical power limitation (<100 segregants).

We did not test additional wild backgrounds, but one could very well apply a similar protocol to many divergent strains and compare their morphological distance to their genetic or transcriptomic divergence. Such a study may indicate whether morphological differences co-evolved with genetic or regulatory divergence, or if they were driven by other differences such as environmental conditions.

One of the challenging aims of modern and future quantitative genetics is the simultaneous dissection of multiple traits. For example, clinical phenotypes are now collected systematically from cohorts of individuals, and molecular phenotypes such as biochemical dosage or gene expression profiling can be included in mapping strategies [14,22]. We show here that yeast cellular morphology represents a large set of quantitative phenotypes with complex inheritance. Although we applied a very basic mapping strategy involving single markers and single traits, the dataset presented here can provide a model framework for development and evaluation of mapping methodologies optimised for multiple traits [12,23]. In particular, we showed that many morphological phenotypes could be correlated to the inheritance of gene expression levels. For example, the brightness difference of the cell wall (highest minus lowest concanavalin-A signal along the wall of one cell) was correlated to the expression of *FLO11*, which is involved in cell-surface variation within yeast cultures [24]. In several cases, the gene expression trait in question was previously mapped to an eQTL [16], whereas the morphological trait remained unmapped. This probably results from the higher statistical power of Brem et al. who used 112 segregants instead of 62. This situation is similar to a clinical case where a disease trait is correlated to an expression signature on the basis of few clinical samples, but where genetic variations controlling this signature are mapped from a large-scale study contributed by many human donors. In this context, eQTL are candidate QTL for the correlated trait. We found 224 cases where a morphological trait was significantly correlated to a gene expression level for which Brem et al. identified an eQTL. We re-examined the nominal linkage *p*-values between these loci and the corresponding morphological trait: 92 cases showed *p* < 0.002, which is an acceptable threshold to account for multiplicity when considering only 224 candidate loci instead of the entire genome. Although most of these cases reflected genetic linkages already identified in our genome scan, they suggested eight additional loci. This indirect mapping of QTL via the use of eQTL seemed therefore promising. However, we tried but failed to validate candidate genes at one of these loci by engineering and characterizing strains where alleles were replaced. We consider that further investigation of these eight loci needs to be done before declaring them as valid QTL. The approach is nonetheless likely to be helpful in studies combining eQTL and phenotypic mapping.

Interestingly, several traits that we mapped were not direct measures of cellular features but coefficients of variation of such measures across the sample. In several cases, the measure itself (e.g., its mean) was not linked to the QTL controlling its coefficient of variation. For example, parameter C13_C, which measured the fitness of the mother cell for ellipse, was not mapped and was not correlated to the *GPA1* genotype (*p* = 0.15), whereas its coefficient of variation was mapped to *GPA1*, and this mapping was further validated by the *gpa1-I469S* strain (*p* = 0.01). This argues that the *gpa1I469S* polymorphism does not affect the shape of all cells but rather the fraction of cells that are elliptic. These observations imply that genetic variation can affect cell-to-cell variability of cellular traits, without necessarily influencing the mean trait itself. This is particularly important when considering genetic susceptibility to common human or animal phenotypes. If genotypes affect the distribution of a phenotype among “identical” cells of a tissue, such genotypes are likely to modulate the phenotype penetrance. It is therefore tempting to propose a nondeterministic view of genetic predisposition, whereby incomplete penetrance does not only result from environmental exposures but also from levels of cell-to-cell heterogeneity.

We showed that the natural polymorphisms affecting yeast morphological traits do not preferentially reside in genes in which deletions affect these traits. This comparison of two alternative genomic approaches (systematic mutagenesis versus QTL mapping) was possible because both studies were performed on the same platform. We conclude that these two approaches are complementary for the functional study of genomes of model organisms. This is important since both approaches are widely used and heavily funded [25,26]. There are at least four possible explanations for this complementarity between the two approaches. First, unlike QTL mapping, the deletion approach does not interrogate essential genes. Second, deletion mutations may have dramatic phenotypic consequences as compared to natural polymorphisms. For example deletion of *RAD50* (YNL250W) results in accumulation of large budded cells arrested or delayed in G2/M because of the failure to repair damaged DNA, but one can imagine that natural sequence variations in the gene might provoke more subtle alterations in the DNA repair system. Third, RM polymorphisms might provide gain-of-function alleles as compared to BY. In this case, the effects are likely to differ from the consequences of a null mutation. Finally, the QTL approach is far from exhaustive because it only interrogates genes containing functional polymorphisms between the backgrounds considered. We also note that in our case, although genome annotation was essential to characterize the effect of *GPA1* alleles, data from systematic mutagenesis could not provide candidate QTL. The power of the candidate-gene approach for QTL mapping has been debated [27], and a previous study on yeast-sporulation efficiency illustrated how functional annotations of some genes poorly explained their QTL effect [2]. It is, therefore, essential to mix information from multiple sources to define candidates and to maintain efforts on genome scans that are free of hypotheses.

## Materials and Methods

### Strains.

Strains used were BY4716 [28] and YEF1946, which is isogenic to RM11-1a [16]. BY-*gpa1I469S* [21] and BYxRM segregants [16] were kindly provided by L. Kruglyak together with their genotypes and transcriptome data.

### Acquisition of quantitative cellular morphological traits.

Microscopic observation and data processing were essentially similar as previously described [15]. Briefly, cultures were grown to 1 × 10^7^ cells/ml in synthetic C medium at 30 °C. Cells were fixed with 3.7% formaldehyde and stained with rhodamine-phalloidine, fluorescein isothiocyanate-concanavalin A, and DAPI. Cells were observed using an Axioplan 2 microscope with 100× Plan-neofluar objective lens (Carl Zeiss, http://www.zeiss.com). Digital images were acquired with CoolSNAP cooled-CCD camera (Roper Scientific, http://www.roperscientific.com) and MetaMorph Software (Molecular Devices, http://www.moleculardevices.com). Images were processed with CalMorph [15] to generate quantitative parameters (or traits) of yeast morphology. A minimum of 200 cells was analysed by culture. For natural variation of S. cerevisiae, nine cultures for either BY4716 or YEF1946 strain were used. For QTL mapping, three cultures for each segregant were used.

### Comparison of measurement errors of DNA-related versus other traits.

For every trait, its coefficient of variation across the 126 replicated cultures of the wild-type strain described in Ohya et al. [15] was computed. The 272 DNA-related traits had a mean coefficient of variation of 15% while the mean of all the others was of 29%, and the difference was significant (*p* = 0.015, Wilcoxon Mann-Whitney).

### Genetic segregation analysis and QTL mapping.

All statistical analyses were made using purpose-developed C codes or using R software (http://www.r-project.org). Heritability was measured as (VarS − VarE)/VarS, where VarS is the variance among segregants, and VarE is an estimate of the environmental variance calculated on the parental replicates. Since three independent phenotypic values were available for each segregant, the variance among segregants was computed three times on independent series. VarS was estimated by the average of these three variances.

For each of the 501 traits, transgressive segregation was tested as in [16], except that the procedure was applied three times, one for each independent series of segregant values. The statistic used was the number *j* of segregants showing a phenotype at least 2σ higher than the mean phenotype of the highest parent, or at least 2σ lower than the mean phenotype of the lowest parent, where σ was the pooled standard deviation of parental replicates. To infer significance, for each trait all parental and segregant values were pooled together and were reassigned to null parents and null segregants at random from this pool. The total number of such null traits with *j* greater than a given threshold *j*_0_ was the expected number of false positives, *N*_false_(*j*_0_). FDR was the *N*_false_(*j*_0_)/*N*_actual_(*j*_0_) ratio, where *N*_actual_(*j*_0_) was the number of real traits showing *j* > *j*_0_. The estimated total number of transgressive traits, *N*_actual_(*j*_0_) − *N*_false_(*j*_0_), was highest for *j*_0_ = 6 and was 157, 128, and 124 in the three replicated series of segregants data, respectively. This corresponded to one-fourth of the total number of traits. At *j*_0_ = 18, FDR was <0.05 and 34 traits were significant in all three series (Table S2).

Similarly, on each series of independent segregant data, we used the test described in Brem et al. [16] to assign *p*-values of epistasis to each trait. Highest *N*_actual_ − *N*_false_ values were 200, 207, and 221 traits in the three series, respectively, corresponding to two-fifths of the total number of traits. At *p* < 0.007, FDR was <0.05, and 98 traits were significant in all three series (Table S3).

For each trait with heritability greater than 50%, the genome was scanned for QTL as follows. Triplicates were averaged to give a single phenotypic value for each segregant. Marker–trait association was tested using the Wilcoxon Mann-Whitney test, and significance was assessed by permutation test as described previously [5]. At a given nominal *p*-value threshold, the FDR was computed as the ratio between the expected number of false positive counts (number of traits that could be mapped after permuting segregant index, averaged across 100 permutations) and the number of traits with detected linkage.

Since all tests were nonparametric, we worked on all 501 traits without distinguishing the set of 254 traits that were previously determined to fit normality (called “reliable” in Ohya et al. 2005) [15].

### Inference of correlations between morphological traits and gene expression.

To test for association between expression levels (5,740 genes) and traits (247 traits with heritability >50%), Pearson correlation coefficients *R* were computed for each pair of messenger level and morphological trait. For a given *R*_0_ cutoff, the number of traits showing |*R*| > *R*_0_, with at least one expression level, was computed after permuting the segregant indexes. This number was averaged across 100 permutations. FDR was then determined as the ratio between this number of expected false positives and the number of traits correlated to expression levels prior to permutation. To mine for traits/GO-terms associations, we clustered the 103 genes and 104 traits correlated at FDR = 0.1 and visually examined the correlation map for regions enriched in genes/traits correlations (Figure 4). Gene lists of each of the four territories described in text were piped in the GO Term Finder (http://db.yeastgenome.org/cgi-bin/GO/goTermFinder) to infer significance of GO term enrichments.

### Comparison of QTL to gene-deletion results.

We segmented the genome into 20-kb bins and assigned each of the 67 traits mapped at FDR = 0.05 to the bin showing highest genetic linkage (as in Table 2). Traits linking to both Chromosomes XIV and XV were assigned to both places by choosing the best bin on each chromosome. This way, 13 bins were linked to traits, with the number of traits per bin ranging from one to 21. For each bin, we considered all genes located within 20 kb of the center of the bin and asked whether their deletions affected one of the trait linked to the bin (at *p* < 0.0001 in the Ohya et al. 2005 dataset). This was the case for only one of the 13 bins. This search involved a high number (2,863) of gene/trait combinations, and finding one positive bin could therefore result from chance only. To test this, we reassigned the 13 bins to random places on the genome and re-examined it to determine if deletions of genes in their vicinity could explain one of their linked traits. We ran this test five times and obtained hits for zero, two, zero, three, and two bins at the respective runs. Thus, obtaining one positive bin in the actual data was not statistically significant.

## Supporting Information

Table S1 Description of 501 Parameters (102 KB XLS)

Table S2 List of Transgressive Parameters at FDR = 0.05 (21 KB XLS)

Table S3 List of Parameters Showing Epistasis at FDR = 0.05 (24 KB XLS)

Table S4 Linkage Results (37 KB XLS)

Table S5 Genetic Correlations between Morphological Traits and Gene Expression Levels at FDR = 0.1 (47 KB XLS)

## Abbreviations

BY: BY4716
eQTL: QTL controlling gene expression
FDR: false discovery rate
GO: gene ontology
QTL: quantitative trait loci
RM: YEF1946
